# Bifidobacteria and Celiac Disease: Mechanisms of Probiotic Action in Reducing Gluten‐Induced Cytotoxicity and Inflammation

**DOI:** 10.1002/mnfr.70222

**Published:** 2025-08-31

**Authors:** Taynara Cipriano Scherer, Ivan De Marco, Natália Regina Coldebella Ferreira, Tatiana Colombo Pimentel, Marciane Magnani, Guilherme de Souza Hassemer, Amanda Bagolin do Nascimento, Silvani Verruck

**Affiliations:** ^1^ Department of Pharmacy Health Sciences Center Federal University of Santa Catarina Santa Catarina Brazil; ^2^ Department of Food Science and Technology Agricultural Sciences Center Federal University of Santa Catarina Santa Catarina Brazil; ^3^ Federal Institute of Science and Technology of Paraná (IFPR) Paranavaí Brazil; ^4^ Laboratory of Microbial Processes in Foods Federal University of Paraiba João Pessoa PB Brazil; ^5^ Department of Nutrition Health Sciences Center Federal University of Santa Catarina Santa Catarina Brazil

**Keywords:** celiac disease, gluten, mechanisms, probiotics

## Abstract

Celiac disease (CD) is an immune‐mediated systemic disorder triggered by gluten peptides present in the diets of genetically susceptible individuals, leading to a range of intestinal and extra‐intestinal manifestations. Although managed by a gluten‐free diet (GFD), symptoms persist in 30%–50% of treated individuals despite apparent dietary compliance. Accordingly, the present review explores how bifidobacteria may mediate cytotoxic and proinflammatory responses induced by gluten‐derived peptides, contributing to the modulation of CD symptoms. Experimental in vitro studies, primarily using Caco‐2 cells and immune cell models, have shown that strains such as *Bifidobacterium longum* IATA‐ES1, *Bifidobacterium lactis*, *Bifidobacterium bifidum* IATA‐ES2, and *B. lactis* Natren Life Start super strain (NLS‐SS) can induce COX‐1 expression and reduce COX‐2, inhibit zonulin release, degrade gliadin‐derived peptides, and suppress CXCR3 mRNA expression and inflammatory mediators (e.g., TNF‐α, IFN‐γ, NF‐κB, and IL‐1β). Animal studies have provided evidence of immunomodulatory effects and improved mucosal responses, while human clinical trials have reported improvements in gastrointestinal symptoms and inflammatory markers with probiotic interventions. These findings support the potential of *Bifidobacterium* spp. as adjunctive agents in CD management. However, further clinical research is needed to clarify strain‐specific effects and confirm the translational relevance of these mechanisms.

## Graphical abstract

Celiac disease (CD) is triggered by gluten in genetically predisposed individuals and is mainly treated with a gluten‐free diet. However, symptoms may persist. This review explores how certain *Bifidobacterium* strains may reduce inflammation and gluten toxicity through six mechanisms, including modulation of immune responses and gut microbiota. These findings highlight their potential as supportive therapy for managing CD.

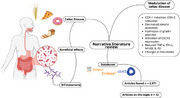

**Abbreviations**
Caco‐2 cells: intestinal cell model
CCR5: C‐C motif chemokine receptor 5
CD: celiac disease
CD4/CD28/CD40/CD80/CD86/CD152: costimulatory and regulatory molecules on T cells and antigen‐presenting cells
CFU: colony‐forming unit
COXs: cyclooxygenases
CTLA‐4: cytotoxic T‐lymphocyte‐associated protein 4
CXCR3: C‐X‐C motif chemokine receptor 3
EGFR: epidermal growth factor receptor
GALT: gut‐associated lymphoid tissue
GFD: gluten‐free diet
GSRS: Gastrointestinal Symptom Rating Scale
HLA‐DQ2/HLA‐DQ8: human leukocyte antigen types DQ2 and DQ8
IBS‐SSS: irritable bowel syndrome severity scoring system
IFN‐γ: interferon gamma
IgA DGP: immunoglobulin A against deamidated gliadin peptide
IgA tTg: immunoglobulin A against tissue transglutaminase
IL‐1β/IL‐10/IL‐12/IL‐15: interleukins
JAMs: junctional adhesion molecules
MHC II: major histocompatibility complex class II
MIP‐1β: chemokine agonist
Mrna: messenger ribonucleic acid
NF‐κB: nuclear factor kappa B
NK: natural killer cells
PBMCs: peripheral blood mononuclear cells
PKC‐α: protein kinase C alpha
PT‐BSA: peptic‐tryptic digest of bovine serum albumin
T cells: T lymphocytes
TNF‐α: tumor necrosis factor alpha

## 1 Introduction

Gluten is a complex of storage proteins found in cereals such as wheat, barley, and rye, mainly composed of prolamins and glutelins [1]. Celiac disease (CD) is a chronic immune‐mediated enteropathy triggered by gluten ingestion in genetically predisposed individuals [2]. Genetic predisposition to CD is primarily associated with the human leukocyte antigen (HLA‐DQ) system, specifically the HLA‐DQ2 and HLA‐DQ8 variants, which have an affinity for gliadin peptides and promote immune activation and autoimmunity [3]. In CD, gluten is incompletely digested due to inefficient intestinal proteases, increasing the presence of immunogenic peptides. These peptides cross the permeable intestinal barrier, are deaminated by tissue transglutaminase (tTG), and are presented to CD4+ T cells via HLA‐DQ2/DQ8, triggering Th1/Th17 responses. This leads to inflammation, epithelial damage, villous atrophy, and dysbiosis, worsening the disease [4, 5]. Although the gluten‐free diet (GFD) remains the only established treatment for CD [6, 7], a wealth of scientific evidence has investigated the role of gut microbiota in disease modulation. In this context, probiotics have been studied as adjunctive approaches that may support mucosal healing and reduce residual inflammation in patients adhering to a GFD [8].

In vitro and animal model studies have demonstrated the beneficial effects of *Lactobacillus* and *Bifidobacterium* strains on gliadin peptide digestion, intestinal barrier integrity maintenance, and immune response modulation, as these two genera are natural inhabitants of the digestive tract and can act synergistically [9]. The probiotic effect of these groups is efficient, although strain‐dependent. For instance, *Lacticaseibacillus rhamnosus* GG was able to reduce alterations in intercellular junction proteins and attenuate gliadin‐induced enteropathy in sensitized rats [10], while *Bifidobacterium longum* CECT 7347 demonstrated a similar effect by protecting the intestinal mucosa in animals exposed to gliadin [11]. Similarly, *B. longum* CECT 7347 and *Lacticaseibacillus casei* ATCC 9595 exhibited comparable effects in reducing intestinal inflammation, decreasing TNF‐α levels, and promoting mucosal repair [12, 13] contributing to improved intestinal barrier function.

Although direct comparisons between the mechanisms of action of *Lactobacillus* and *Bifidobacterium* in CD remain limited, studies suggest that bifidobacteria may exert effects equivalent or even superior to lactobacilli, particularly regarding anti‐inflammatory responses [5, 14, 15, 16]. Thus, *Bifidobacterium* spp. stands out as Gram‐positive, anaerobic, non‐sporulating, non‐motile, and catalase‐negative bacilli [17]. These bacteria are widely used as probiotics due to their functional efficacy and safety, supported by their history of human consumption and their natural presence in the human intestine. This safety is recognized by regulatory bodies such as the FDA and EFSA, reflected in the GRAS (Generally Recognized as Safe) and QPS (Qualified Presumption of Safety) statuses [18]. Although there are rare reports of infection [19], studies demonstrate that these bacteria do not pose a health risk to healthy individuals [20, 21, 22, 23, 24, 25, 26].

This genus has shown particular promise in the management of CD due to its ability to modulate immune responses and degrade immunogenic gluten peptides. Strains such as *Bifidobacterium bifidum* IATES2, *B. longum* ATCC 15707, *Bifidobacterium lactis*, and *B. longum* IATA‐ES1 have demonstrated potential to reduce inflammation, decrease gluten immunogenicity, restore intestinal microbiota, and alleviate persistent symptoms even in patients on restrictive diets [18, 19, 20]. Considering that 30%–50% of patients still present residual symptoms and that inadvertent gluten exposure is frequent [8, 27], *Bifidobacterium* spp. emerges as a relevant complementary strategy to the GFD [28]. Therefore, this review focuses on the potential of this probiotic group to modulate the cytotoxic and pro‐inflammatory responses associated with CD, highlighting its implications for disease management.

## 2 Method

A narrative synthesis of the literature was conducted to investigate the research question: What mechanisms of action does the *Bifidobacterium* spp. genus exhibits on the proteins that trigger celiac disease (CD)?3 To identify relevant studies, a systematic search was conducted in the following databases: Elsevier's Scopus (SCOPUS), Embase Indexing and Emtree (EMBASE), Web of Science, and the Latin American and Caribbean Health Sciences Literature (LILACS). The search strategy was developed using DeCS (Health Sciences Descriptors) and MeSH (Medical Subject Headings), applying the following keywords and Boolean operators: ((Celiac Disease) OR (Gluten)) AND (*Bifidobacterium*).

The eligibility criteria included experimental studies conducted in vitro or in vivo (in both animals and humans), published between January 2008 and March 2025, written in English, Portuguese, or Spanish, and without geographic restrictions. Studies were considered only if the full text was accessible or available upon request from the author. To enhance the reliability of the findings, the methodological quality of the included studies was evaluated. For in vivo and clinical trials, we considered elements of the Cochrane risk of bias tool, including randomization, blinding, and outcome reporting. For in vitro studies, although no formal tool is universally adopted, criteria such as cell model relevance, experimental replication, and reporting of quantitative outcomes were assessed. However, a formal GRADE assessment was not applied due to the heterogeneity and predominantly preclinical nature of the included studies.

Exclusion criteria encompassed studies involving non‐adult individuals or non‐adult animals (such as neonates, children, or elderly subjects), research focusing on non‐celiac individuals, and articles exploring *Bifidobacterium* spp., mechanisms in diseases unrelated to CD.

All cited clinical studies involving human participants were conducted in accordance with the principles of ethical research. The original publications reported having obtained institutional ethics approval and informed consent, ensuring compliance with international research ethics standards. Additionally, all in vivo studies using animal models also reported prior approval by institutional animal ethics committees, in accordance with guidelines for animal research.

## 3 Result

**FIGURE 1:**
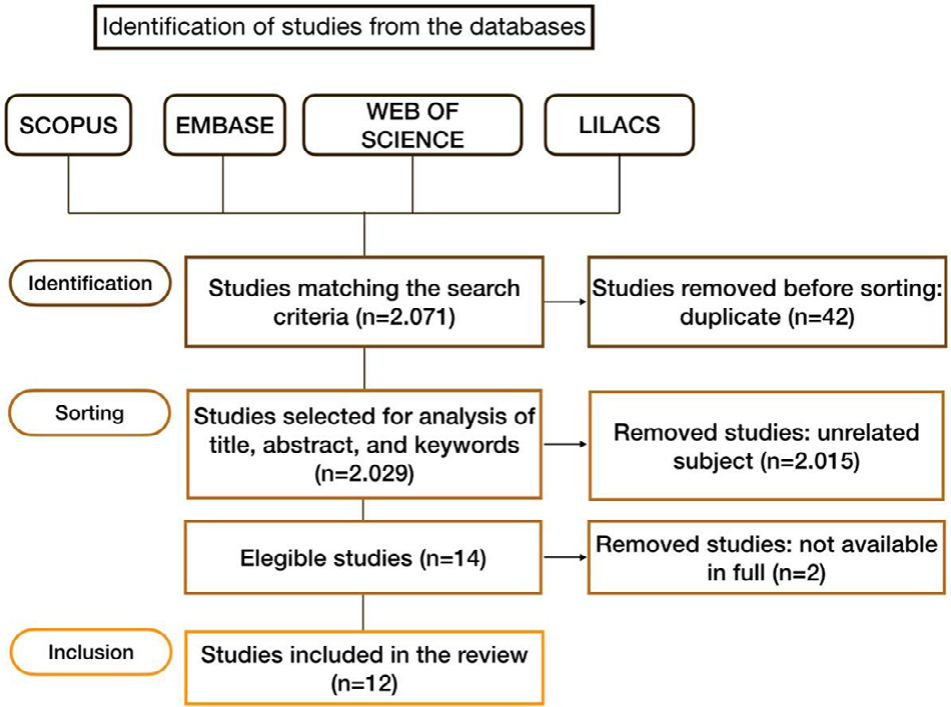
Flowchart of the article selection process.

Figure 1 shows a flowchart of the article selection process. Initially, 2071 scientific productions were retrieved. These results were imported into the Mendeley reference manager, and subsequent steps—including the exclusion of 42 duplicate articles—were conducted using this platform. Upon reviewing titles, abstracts, and keywords, 2027 documents were excluded due to non‐compliance with inclusion criteria or alignment with exclusion criteria. Another seven studies were eliminated as they were out of scope, and two because they involved non‐adult subjects. Additionally, two studies lacked full access to their complete documents, despite their relevance to the topic. Finally, 12 studies were included in this review.

## 4 Discussion

### 4.1 Immunological and Physiological Mechanisms of CD

A singular layer of epithelial cells in the human intestine forms the primary interface between the host and its environment. The complex arrangement of the intestinal mucosa emphasizes sophisticated communication between the epithelial cells and the underlying immune system, which is crucial for the coordinated surveillance of luminal contents. This mucosa plays a vital role in balancing nutrient absorption, fluid secretion, and protection against microorganisms, toxins, and food antigens. The cohesion of epithelial cells is ensured by tight junctions, adherens junctions, and desmosomes [29].

As outlined by Otani and Furuse [30], tight junctions, situated apically between neighboring epithelial and endothelial cells, constitute a permeability barrier that controls macromolecule diffusion through the intercellular space (gate function). They also segregate apical and basolateral plasma membrane domains (fence function) [31]. Comprising transmembrane proteins such as claudins, tight junctions play a crucial role in regulating paracellular permeability. Claudins, occludins (tight junction proteins), junctional adhesion molecules (JAMs), tricellulin, and angulins form these tight junctions. These proteins interact through both homophilic and heterophilic interactions, linking with intracellular scaffold proteins, such as occludins, which are anchored to the actin cytoskeleton. Their cooperation maintains tight junction integrity and governs macromolecule passage [32, 33].

Fasano [34] and Sturgeon and Fasano [35] described the role of zonulin in regulating intestinal permeability and its association with chronic inflammatory disorders, including CD. After gluten ingestion, gliadin activates zonulin signaling in CD patients, altering tight junctions and increasing intestinal permeability. As a result, gluten‐derived peptides can reach the lamina propria (mucosa) via transcellular or paracellular pathways. There, tTG modifies these peptides, enhancing their affinity for major histocompatibility complex (MHC II) molecules and making them toxic and immunogenic in HLA‐DQ2 or DQ8‐positive patients [36]. Proline residues make gluten‐derived peptides preferred substrates for tTG, whose modifications occur through deamidation or transamidation [37].

Peptides presented by HLA‐DQ2/DQ8 protein subunits on dendritic cell surfaces to gluten‐reactive T cells induce innate and adaptive responses. This involves IFN‐γ and IL‐15 production, culminating in the CD pathogenic process. This sequence triggers immune‐mediated enteropathy, leading to intestinal inflammation, villous atrophy, crypt hyperplasia, intraepithelial lymphocyte infiltration, chronic diarrhea, and weight loss. The cross‐link between gliadin and tTG generates new epitopes that activate the innate immune response, yielding autoantibodies against tTG [38]. Probiotics have been identified for their ability to enhance the human digestive system via diverse mechanisms, such as inhibiting pathogen growth, competing for nutrients, modulating the immune system, and protecting epithelial cells against toxic gliadin peptides [39].

Moreover, a key immunological mechanism involved in CD is the activation of the nuclear factor kappa B (NF‐κB) signaling pathway. Gluten‐derived peptides can trigger NF‐κB activation in intestinal epithelial cells and antigen‐presenting cells, such as monocytes and dendritic cells, promoting the transcription of pro‐inflammatory cytokines, including tumor necrosis TNF‐α, interleukin‐1β (IL‐1β), and chemokines [13, 40, 41]. This contributes to the recruitment of inflammatory cells to the intestinal mucosa and the perpetuation of tissue inflammation. NF‐κB activation has been recognized as a central element mediating the inflammatory cascade characteristic of CD, correlating with increased intestinal permeability and epithelial barrier dysfunction [40, 41].

### 4.2 Action Mechanisms of *Bifidobacterium* spp. on CD

**FIGURE 2:**
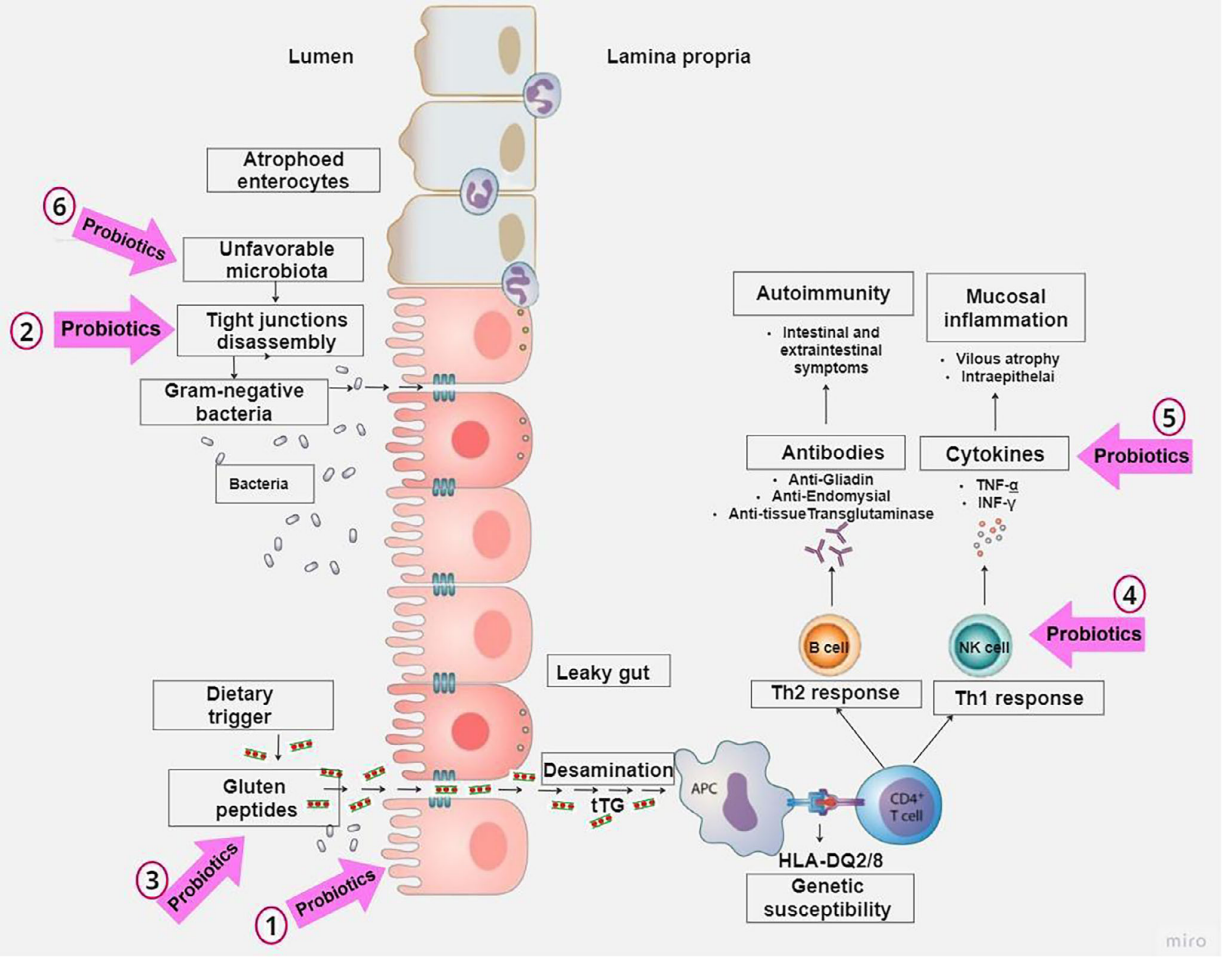
Possible pathways of *Bifidobacterium* spp. action in CD, including the mechanisms of action. (1) induction of COX‐1 expression in Caco‐2 cells and reduction of COX‐2 expression, ensuring intestinal mucosa integrity, (2) decreased zonulin activation and inhibition of CXCR3 mRNA expression, preventing tight junction breakdown, (3) hydrolysis of gliadin‐derived peptides, preventing excess gluten peptides in the intestinal lumen, (4) and (5) control of the Th1 response and reduction of TNF‐α, IFN‐γ, NF‐kB, and IL‐1β production, (6) alteration of the dysregulated intestinal microbiota in CD.

The potential mechanisms of action of *Bifidobacterium* spp. have been elucidated from the pathogenic process of CD. A summary of the mechanisms by which probiotics of the *Bifidobacterium* genus act based on the studies included in the review can be found in Table 1. Figure 2 illustrates the sites of action of this genus, which were addressed in the selected studies.

**TABLE 1:** Summary of the mechanisms by which *Bifidobacterium* acts against CD.

Type of study	Strains evaluated	Administered dosage	Duration of intervention	Action mechanism	Reference
In vitro experimental model using human caco‐2 cells	*Bifidobacterium lactis* and *L. fermentum*	∼ 10^7^ CFU/mL	N.A	Induction of COX‐1 expression in Caco‐2 cells and reduction of COX‐2 expression. Decreased activation of zonulin.	Lindfors et al. [5]
In vitro experimental model using human caco‐2 cells	*Bifidobacterium longum* IATA‐ES1, *Bifidobacterium bifidum* IATA‐ES2 and *B. animalis* IATA‐A2	∼ 10^8^ CFU/mL	N.A	Hydrolysis of gliadins‐derived peptides. Reduced production of TNF‐*α*, INF‐*γ*, NF‐kB, and IL‐1*β*. Inhibition of CXCR3 mRNA expression.	Laparra and Sanz [41]
In vitro experimental model (PBMCs + Caco‐2 cells)	*B. bifidum* IATA‐ES2, *B. longum* ATCC15707	∼ 10^6^ CFU/mL	N.A	Decreased production of TNF‐*α*, INF‐*γ*, NF‐kB, and IL‐1*β*.	De Palma et al. [28]
In vitro experimental model using human caco‐2 cells	*B. bifidum*, *B. longum*, *B. breve* and *B. animalis*	∼ 10^9^ CFU/mL	N.A	Decreased production of TNF‐*α*, INF‐*γ*, NF‐kB, and IL‐1*β*.	De Almeida et al. [60]
In vitro experimental model using human caco‐2 cells	*L. paracasei* 101/37 LMG P‐17504, *L. plantarum* 14 D CECT 4528, *B. animalis subsp. lactis* Bi1 LMG P‐17502, *B. breve* Bbr8 LMG P‐17501 and *B. breve* BL10 LMG P‐17500	∼ 10^10^ CFU/mL	N.A	Hydrolysis of gliadin‐derived peptides.	Giorgi et al. [47]
In vitro experimental model using human caco‐2 cells	*B. longum* (ATCC 15708), *L. acidophilus* (ATCC 4356) e *L. plantarum* (ATCC 8014)	∼ 10^9^ CFU/mL	N.A	Hydrolysis of gliadin peptides and suppression of gliadin‐induced inflammatory responses in Caco‐2 cells.	Ramedani et al. [67]
Animal model using newborn female Wistar rats	*B. longum* CECT 7347	∼ 10^9^ CFU/mL	10 days	Hydrolysis of gliadins‐derived peptides. Reduced production of TNF‐*α*, INF‐*γ*, NF‐kB, and IL‐1*β*.	Laparra et al. [13]
Animal model using male mice	* B. longum *CCFM1216 *B. longum* JCM1217 *B. logum* CCFM1218	∼ 10^9^ CFU/mL	10 weeks	Reduction in zonulin release; Decrease in IL‐15 levels in the duodenum	Wang et al. [68]
Randomized, double‐blind, placebo‐controlled study in 22 adults with CD	*B. infantis* NLS Super Strain	∼ 10^9^ CFU/mL (t.i.d)	3 weeks	Alteration of inflammatory factors such as MIB‐1B and CC5.	Smecuol et al. [61]
Randomized, crossover, double‐blind, placebo‐controlled study in 12 adults with CD (GFD ≥2 years) and persistent symptoms	*B. infantis* NLS Super Strain	∼ 10^9^ CFU/mL (t.i.d)	3 weeks	Changes in gut microbiome.	Smecuol et al. [63]
Randomized, double‐blind, placebo‐controlled trial in 45 adults with CD (GFD ≥12 months) and persistent symptoms	*B. longum*, *B. infantis*, *B. breve*, *L. plantarum*, *L. paracasei, L. acidophilus*, *L. delbrueckii* subsp. *bulgaricus* and *S. thermophilus*,	∼ 10^11^ CFU/mL (b.i.d)	12 weeks	Changes in gut microbiome.	Harnett et al. [62]
Multicenter, randomized, double‐blind, placebo‐controlled trial conducted in 109 adults with CD on GFD (≥2 years) presenting IBS‐type symptoms	*L. casei* LMG 101/37 P‐17504, *L. plantarum* CECT 4528, *B. animalis* subsp. *lactis* Bi1 LMG P‐17502, *B. breve* Bbr8 LMG P‐17501, *B. breve* Bl10 LMG P‐17500	∼ 10.60	6 weeks	Changes in gut microbiome.	Francavilla et al. [64]

Abbreviations: b.i.d, twice a day (*bis in die*, Latin); t.i.d, three times a day (*ter in die*, Latin); N.A., Not applied.

#### 4.2.1 Induction of COX‐1 Expression in Caco‐2 Cells and Reduction of COX‐2 Expression

Cyclooxygenases (COXs) are conserved enzymes with two primary forms, COX‐1 and COX‐2, resulting from distinct gene coding. These enzymes jointly generate an unstable prostaglandin endoperoxide, PGH2, from arachidonic acid [42]. COX‐1 produces prostaglandins crucial for sustaining regular mucosal integrity, while COX‐2 is linked to states of inflammation [43].

Lindfors et al. [5] in an experimental study using an in vitro model system with human intestinal epithelial cell line Caco‐2, associated the *B. lactis* potential to counter gliadin‐induced damage by inducing COX‐1 expression in Caco‐2 cells and concurrently suppressing pro‐inflammatory COX‐2 expression. This action hints at an improved prognosis for CD. However, this proposed mechanism was observed exclusively in vitro, and its relevance in in vivo settings remains to be confirmed. Thus, while promising, the effect of *B. lactis* on COX expression is not yet well established in the broader literature. Further investigations are imperative to establish a more precise understanding of *B. lactis*' impact on these enzymes. Subsequent studies could shed light on the intricacies of this interaction using animal models and human clinical trials to clarify the therapeutic potential and mechanistic pathways through which *B. lactis* may exert protective effects in coeliac disease.

#### 4.2.2 Reduction in Zonulin Activation

As previously discussed, zonulin plays a central role in regulating intercellular junctions in the intestine, with its exacerbated activation being a determining factor in the increased intestinal permeability observed in individuals susceptible to CD.

**FIGURE 3:**
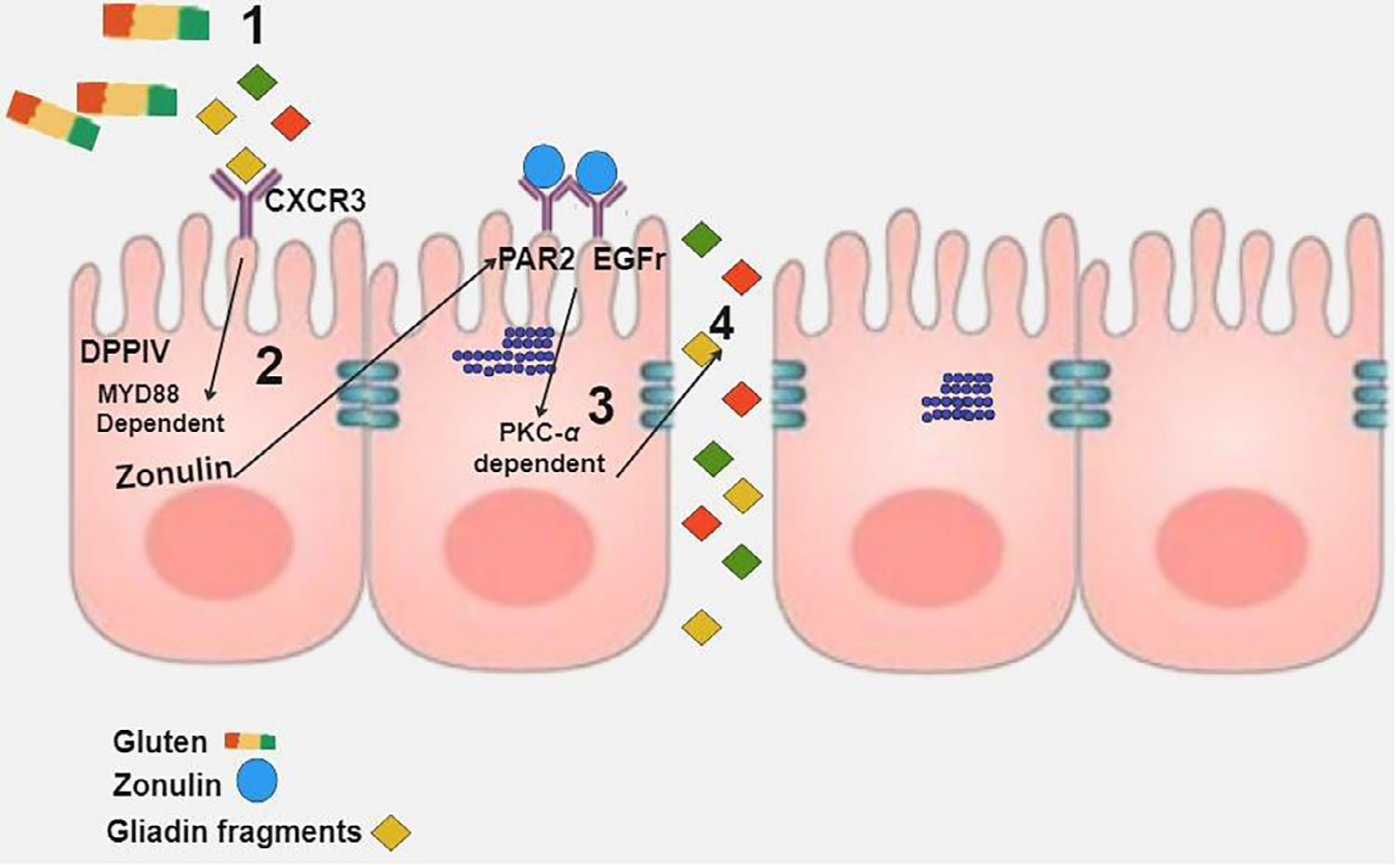
Zonulin release mechanism from gluten ingestion: (1) Specific gliadin peptides, (2) CXCR3, (3) PKC‐α, (4) non‐self antigens.

Gliadin acts as a trigger for zonulin release. In individuals susceptible to it, particular non‐digestible gliadin peptides can attach to the CXCR3 receptor located on the apical surfaces of enterocytes. Subsequent MyD88‐dependent zonulin release activates the epidermal growth factor receptor (EGFR) through protease‐activated receptor 2 (PAR2). This prompts protein kinase C (PKC‐*α*)‐dependent tight junction degradation, enhancing intestinal permeability. Consequently, non‐self antigens pass paracellularly to the lamina propria, engaging with the immune system (Figure 3) [35].

The study conducted by Lindfors et al. [5] highlighted the potential of the *Bifidobacterium* genus in modulating this process, demonstrating its ability to reduce zonulin release and preserve cytoskeletal organization and intestinal permeability in in vitro models. The research used the human intestinal epithelial cell line Caco‐2 to evaluate the protective effects of *Limosilactobacillus fermentum* and *B. lactis* against gliadin peptide‐induced cellular damage. The evaluation included transepithelial resistance, actin cytoskeleton configuration (quantified by the degree of membrane ruffling), and zonulin protein expression.

Lindfors et al. [5] showed that *B. lactis* was able to attenuate the increase in epithelial permeability triggered by gliadin. At the concentration of 10^6^ CFU/mL, *B. lactis* provided partial protection, while at the highest concentration tested (10^7^ CFU/mL), full protection against changes in transepithelial resistance was achieved. In addition, the strain inhibited the formation of membrane ruffling in Caco‐2 cells caused by gliadin administration. When supplemented with 10^7^ CFU/mL of *B. lactis*, the cultures exhibited a percentage of membrane ruffling comparable to the control (PT‐BSA). The modulatory effect on zonulin was particularly relevant: *B. lactis* contributed to normalizing the expression of this protein, counteracting the deleterious effects of gliadin. Although these results are promising, the use of the in vitro model limits direct extrapolation to in vivo conditions. The complexity of the human gastrointestinal environment, including microbial diversity and immune factors, may interfere with the observed effects. Thus, although the role of probiotic dosage in achieving the desired effects was evident, additional studies, including long‐term clinical trials, are necessary to validate the practical applicability and safety of using *Bifidobacterium* strains as adjunctive therapy in CD.

Among the articles analyzed in this review, the study by Lindfors et al. [5] was the only one to present a direct comparison between a representative of the *Lactobacillus* group (*L. fermentum*) and a *Bifidobacterium* strain (*B. lactis*). Among the strains evaluated, *B. lactis* demonstrated superior performance. Although both strains exhibited protective effects against gliadin‐induced epithelial damage, *B. lactis* was notably more effective in restoring transepithelial resistance, inhibiting membrane ruffling, and normalizing zonulin expression. Particularly at the concentration of 10^7^ CFU/mL, *B. lactis* achieved full protection against barrier dysfunction, whereas *L. fermentum* provided only partial protection under similar conditions. These findings suggest that *B. lactis* may represent a more promising candidate for therapeutic strategies aimed at preserving intestinal barrier integrity in CD.

#### 4.2.3 Probiotic‐Assisted Hydrolysis of Gluten Peptides

When considering the main dietary proteins, gluten is the only one that contains 15% proline residues and 35% glutamine. The high concentration of glutamine, especially proline, prevents complete degradation by human gastric and pancreatic enzymes, resulting in oligopeptides in the small intestine that are resistant to further proteolysis and toxic to individuals with CD [44, 45]. Within this framework, several investigations have examined the potential of probiotics, including *Bifidobacterium* species, as an alternative for hydrolyzing gluten peptides that are not degraded in individuals with CD [13, 41, 46, 47].

Gliadin‐derived peptides with specific amino acid sequences stimulate proinflammatory cellular responses in enterocytes and immunocompetent cells through the CXCR3 receptor‐associated chemokine receptor signaling pathways [48]. Experimental models have shown that gliadin‐derived peptides perpetuate inflammatory signaling, contributing to the release of cytokines like TNF‐α and IL‐1β [37, 49].

Giorgi et al. [47] conducted an in vitro study assessing a blend containing *Lacticaseibacillus paracasei* 101/37 LMG P‐17504, *Lactiplantibacillus plantarum* 14 D CECT 4528, *B. animalis* subsp. *lactis* Bil, *Bifidobacterium breve* Bbr8, and *B. breve* BL10 to hydrolyze immunogenic gliadin peptides. Through the application of techniques such as sodium dodecyl sulfate‐polyacrylamide gel electrophoresis (SDS‐PAGE), high‐performance liquid chromatography (HPLC) measurements, and molecular fractionation experiments, the research demonstrated a noteworthy reduction in the molecular size of PT‐gliadin fragments upon treatment with probiotic bacteria. A specific ELISA assay estimated that a greater quantity of peptides with molecular weight below three Kilodalton (kDa) was observed in PT‐gliadin when inoculated with the probiotic mixture, compared to isolated PT‐gliadin. Therefore, the probiotic strains applied in Giorgi et al. [47] decrease the presence of the immunotoxic 33‐mer peptide when it is the exclusive amino acid source. This affirms the existence of a distinct transport system within the selected bacteria for assimilating immunogenic oligopeptides, aligning with observations made for other bacterial strains [50].

A combination of different strains was necessary for gluten peptide hydrolysis, and it was found that the probiotic mixture exhibited characteristics of a combination of different peptidases, contributing to the hydrolysis of the 33‐mer peptide [47]. De Angelis et al. [44] and De Angelis et al. [51] asserted that no single bacterial strain has all the essential peptidases required for the complete hydrolysis of immunogenic gluten epitopes. According to De Angelis [44] at least three peptidases (PepN, PepX, and PepO) are required to hydrolyze the 33‐mer epitope without generating immunogenic‐derived peptides. The research identified a combination of 10 strains that collectively furnished the essential peptidases for the comprehensive degradation of immunogenic gluten peptides, encompassing the 33‐mer associated with CD. Thus, the authors recommended that two lactobacilli strains and three bifidobacteria strains were complementary for the effective hydrolysis of immunogenic epitopes. The low‐molecular‐weight fragments produced following bacterial treatment act to safeguard tight junction proteins, demonstrate antioxidative and anti‐inflammatory properties, and effectively counteract the adverse effects induced by untreated PT‐gliadin.

**FIGURE 4:**
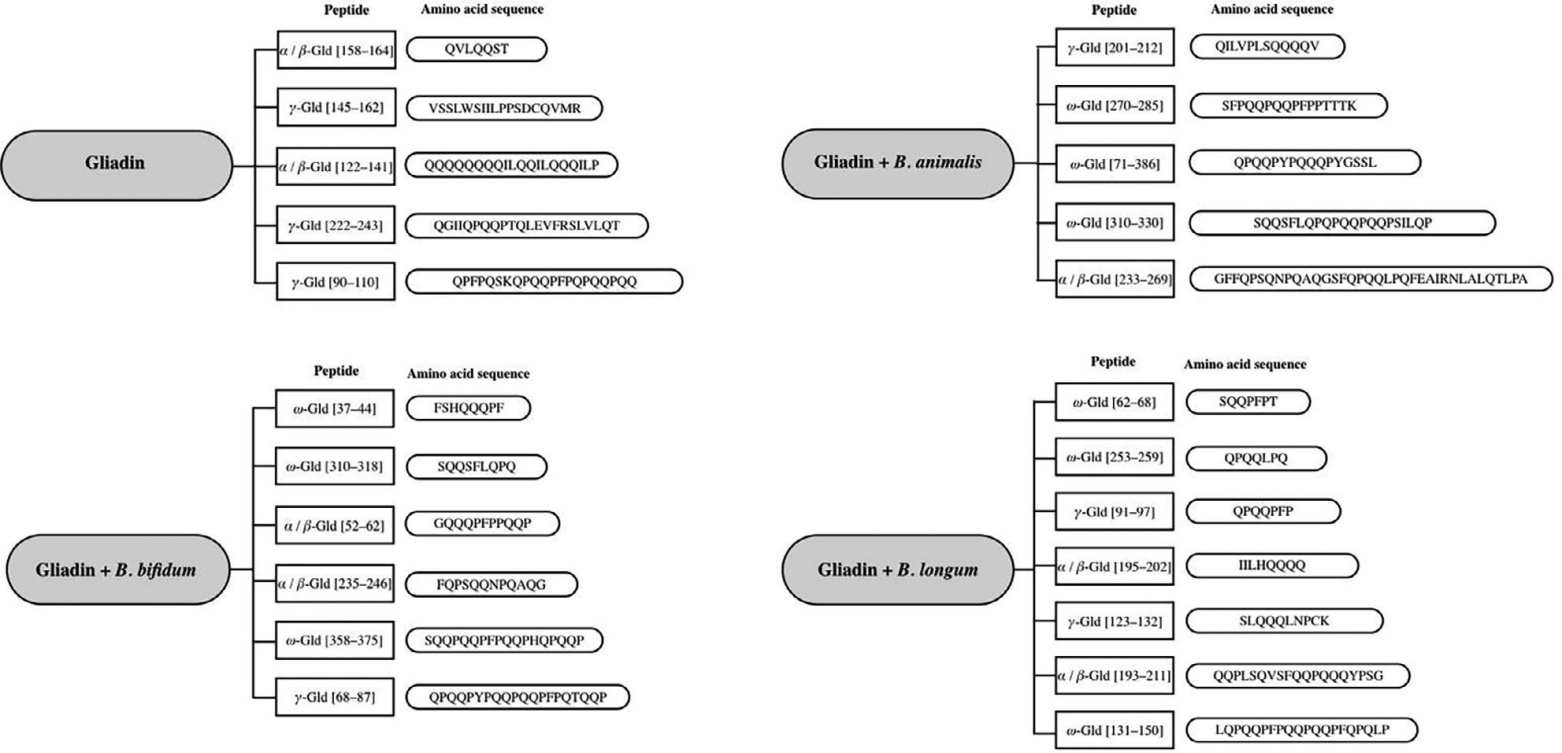
Gliadins‐derived peptides after gastrointestinal digestion with *Bifidobacterium* spp. Adapted from Laparra and Sanz laparra.[41]

During the digestion of gliadin in vitro, the inclusion of specific *Bifidobacterium* strains (*B. bifidum* IATA‐ES2, *B. longum* IATA‐ES1, and *B. animalis* IATA‐A2) produced distinct fragment sequences with lower molecular mass compared to untreated samples, as observed by Laparra and Sanz [41]. This investigation identified peptides resulting from the digestion of non‐inoculated gliadin, including amino acid sequences such as α/β‐Gliadin (122‐141) and α/β‐Gliadin (158–164). As confirmed by Lammers et al. [48] sequences similar to these interact with the CXCR3 receptor, which has implications for cytoskeletal rearrangement, as mentioned earlier. The amino acid sequences identified in the study by Laparra and Sanz [41] were not detected in gliadin digestions inoculated with bifidobacteria (Figure 4). Notably, gliadin digestions inoculated with *B. bifidum*, *B. longum*, and *B. animalis* did not elevate CXCR3 mRNA expression compared to other digested gliadin samples, suggesting a potential role in preserving intestinal barrier integrity. However, inoculated and non‐inoculated gliadin digestions with *B. bifidum* and *B. animalis* exhibited cytotoxic effects on intestinal epithelial cells. In contrast, *B. longum* showed no cytotoxicity, prompting a subsequent investigation [13] that evaluated a specific strain *B. longum* CECT 7347.

Laparra and Sanz [41] evaluated how bifidobacteria mitigate the inflammatory response induced by gliadin peptides. Notably, *B. longum* was the most effective strain in reducing TNF‐α production in Caco‐2 cells, potentially supporting intestinal barrier integrity by limiting cytokine‐mediated tight junction disruption. Additionally, gliadin‐derived peptides activate enterocytes through the CXCR3 chemokine receptor linked to transmembrane G protein, participating in cytoskeletal rearrangement in inflamed tissues and zonulin release [52]. The potential implications of these effects on intestinal barrier function, especially concerning TNF‐α‐induced tight junction‐dependent permeability, are emphasized. However, it is essential to acknowledge the complexity of these interactions within the context of the intricate gastrointestinal environment. The involvement of gliadin‐derived peptides in stimulating enterocytes through the CXCR3 chemokine receptor and its association with cytoskeletal rearrangement and zonulin release adds another layer of complexity to understanding the mechanisms at play. The variation in inhibitory effects produced by bifidobacteria on proinflammatory responses to gliadins in Caco‐2 cells, dependent on the strain considered [41], raises important questions about the specificity and generalizability of these effects. Although *B. longum* exhibited strong inhibitory effects on NF‐kB activation and TNF‐α production triggered by gliadin‐derived peptides additional investigation is needed to ascertain the generalizability of these findings across various strains and under in vivo conditions.

Lastly, Laparra and Sanz [41] suggested that gliadin digestion, when inoculated with *B. bifidum* and *B. longum*, did not elevate CXCR3 mRNA expression in contrast to other digested gliadin samples, potentially aiding in the maintenance of intestinal barrier integrity. Moreover, the decreased TNF‐α production through gliadin digestion inoculated with *B. longum* holds significant physiological implications for CD. TNF‐α and IL‐1β play a crucial role in activating nitric oxide synthase (NOS), considered by Zhao et al. [53] as a mediating enzyme in the interaction between intraepithelial lymphocytes and intestinal epithelial cells, promoting tissue inflammation. Additionally, TNF‐α positively influenced IL‐8 production, a key chemokine attracting inflammatory cells such as neutrophils. Prolonged neutrophil infiltration sustains inflammatory responses, contributing to cellular damage and epithelial barrier dysfunction. The reported findings broaden the spectrum of beneficial effects that probiotic bacteria may confer on intestinal epithelial cell function in CD, warranting further evaluation in individuals with the condition.

Another study focusing on strains of *B. bifidum* and *B. longum* was carried out by De Palma et al. [28] which evaluated the potential immunomodulatory effects of *B. bifidum* IATA‐ES2 and *B. longum* ATCC15707 in comparison to those of intestinal Gram‐negative bacteria (*Bacteroides fragilis* DSM2451, *Escherichia coli* CBL2, and *Shigella* CBD8) in an in vitro model involving peripheral blood mononuclear cells (PBMCs) exposed to gliadin and/or IFN‐γ. This simulation aimed to replicate the conditions of CD in an experimental model. PBMCs served as an in vitro model since blood monocytes continuously replenish mucosal monocytes. Additionally, mucosal dendritic cells accumulate in celiac lesion areas, crucial for activating gluten‐sensitive intestinal T cells recruited from blood monocytes [54]. De Palma et al. [28] observed that *B. bifidum* IATA‐ES2 and *B. longum* ATCC15707 induced a reduced production of Th1‐type cytokines (IFN‐γ and/or IL‐12) while promoting the production of the anti‐inflammatory cytokine IL‐10. This modulation may assist in controlling the Th1‐biased immune response characteristic of CD. Stimulation with *B. bifidum* IATA‐ES2 resulted in diminished TNF‐α production, a factor known, along with IFN‐γ, to increase intestinal epithelial permeability, potentially facilitating the entry of larger antigen loads into the submucosa in CD [54]. The observed effects on TNF‐α production and intestinal epithelial permeability hint at possible implications for the disease. However, translating these in vitro results to CD's complex in vivo environment necessitates caution. Additionally, the multifaceted nature of the immune response in CD involves various factors that may not be entirely mirrored in the simplified in vitro model. Although the study provides a foundation for understanding the immunomodulatory potential of *B. bifidum* IATA‐ES2 and *B. longum* ATCC15707, its applicability to the broader spectrum of CD and its potential therapeutic implications warrants further exploration.

Furthermore, the study focuses on a specific set of strains [28], and the variation in response among different strains of bifidobacteria may not be fully captured. Individual strains exhibited distinct immunomodulatory effects on dendritic cells, and the most pronounced anti‐inflammatory effects were produced by bifidobacteria, which increased IL‐10 production, decreased the expression of co‐stimulatory molecules CD80 and CD40, and decreased IFN‐γ production by T cells. CD40 signaling increases IL‐12 production by dendritic cells, contributing to the Th1‐biased DC phenotype. The upregulation of co‐stimulatory molecules CD80 and CD86 on dendritic cells enhances binding to CD28 and CD152 (CTLA4) on T cells, thereby influencing the type of T cell response and contributing to the Th1 response [55, 56]. Notably, the presence of gliadin did not appear to affect the effects of bacteria on cell surface markers. However, the presence of *Shigella* CBD8 and IFN‐γ heightened the induction of CD40 expression.

As mentioned earlier, the study by Laparra et al. [13] used only the *B. longum* CECT 7347 strain. Unlike previous studies, this research was conducted in an animal model. Intestinal gliadin digestion, including *B. longum* CECT 7347, led to the production of distinct peptide sequences, mitigating their toxic and inflammatory impact on intestinal epithelial cells, as described by Laparra and Sanz [41]. Laparra et al. [13] study marked the initial exploration of the impact of administering a bifidobacterial strain (*B. longum* CECT 7347) during early postnatal life on the intestinal mucosal structure and indicators of both innate and adaptive immunity within an experimental animal model of gliadin‐induced enteropathy. Animal sensitization with IFN‐γ was required, as this inflammatory cytokine appears necessary for causing mucosal damage and immune alterations similar to those observed in human CD [57]. However, sensitization with IFN‐γ alone did not result in histological changes in the study. Hence, the animal model employed in the Laparra et al. [13] study represents an intermediate stage between the proliferative and destructive phases of CD, avoiding complete villous atrophy and disruption of intestinal epithelial integrity as seen in individuals with fully developed CD. Although Laparra et al. [13] investigation provides valuable insights into the potential advantages of *B. longum* CECT 7347 in an animal model, it is crucial to acknowledge the inherent limitations in any animal model's ability to fully mirror the complexities of human CD.

Laparra et al. [13] confirmed that gliadin ingestion in animal models increases pro‐inflammatory markers, including TNF‐α, aligning with observations in humans with CD [40, 41, 58]. However, when administered alone, gliadin did not prompt NF‐kB expression or TNFα production compared to control groups. Gliadin administered independently may trigger a regulatory response, evidenced by the negative regulation of NF‐kB mRNA expression and increased production of IL‐10, promoting tolerance in animals not genetically predisposed to the disease. Additionally, Laparra et al. [13] underscored substantial differences in the immunomodulatory properties of *B. longum* CECT 7347 and *L. casei* ATCC 9595, as the latter strain failed to restore IL‐10 production in the HLA‐DQ8 transgenic mouse model of enteropathy, as demonstrated in another study [12]. IL‐10 production was also stimulated by the administration of *B. longum* CECT 7347 alone in control mice but not TNF‐α, providing additional evidence of the anti‐inflammatory properties of this strain even in the absence of other stimuli, such as gliadin or an inflammatory condition. IL‐10 promotes oral tolerance to dietary antigens by inhibiting chemokine production, facilitating antigen presentation by monocytes and macrophages, and inducing soluble antagonists of proinflammatory cytokines such as IL‐1 and TNFα [59]. Although Laparra et al. [13] study contributes valuable insights into the intricate mechanisms involving NF‐kB, gliadin, and the immunomodulatory properties of specific probiotic strains, a comprehensive understanding of these interactions necessitates further investigation, ideally through human studies and clinical trials.

The study by de Almeida et al. [60], using in vitro models with human cell lines (Caco‐2), evaluated the effect of *B. bifidum*, *B. longum*, *B. breve*, and *B. animalis* species on the digestion of intact gluten proteins (gliadins and glutenins) and the associated immunomodulatory responses induced by the resulting peptides. Upon assessing the cytotoxicity of peptides derived from gluten ingestion, it was noted that peptides digested with gluten in samples lacking *Bifidobacterium* inoculation exhibited a notable cytotoxic response to intestinal epithelial cells. Conversely, peptides obtained from *Bifidobacterium* cultures demonstrated a significant reduction in cytotoxic effects, particularly those generated in the presence of *B. longum*. The decrease in cytotoxic effects in samples from *Bifidobacterium* cultures became more pronounced after 48 h compared to the responses observed in non‐inoculated samples containing gluten peptides (positive controls). This implies that bifidobacteria play a role in mitigating the inactivation of endosomal/lysosomal activities induced by gluten.

De Almeida et al. [60] also examined the immune response to gluten‐digested peptides produced from *Bifidobacterium* cultures, monitoring the production of TNF‐α, (IL)‐1β, and NF‐kB activation associated with innate immune responses. NF‐kB activation is common in the intestinal mucosa of individuals with CD, and the expression of cytokines TNF‐α and IL‐certain gluten peptides positively regulate 1β. Peptides from gluten digestion in non‐inoculated samples activated proinflammatory pathways, triggering NF‐kB (nuclear subunit p65) activation and the production of cytokines (TNF‐α and IL‐1β). In contrast, gluten fragment peptides generated by *Bifidobacterium* cultures led to a reduction in transcription factors and cytokine levels compared to their respective non‐inoculated samples. Moreover, the decrease in TNF‐α expression and NF‐kB activation in *Bifidobacterium* cultures was demonstrated to be species‐dependent.

Considering the alteration of inflammatory factors such as MIP‐1β and CCR5, Smecuol et al. [61] conducted a randomized, double‐blind, placebo‐controlled trial with 22 adult individuals with CD. Participants were randomized to receive two capsules of *Bifidobacterium infantis* NLS super strain (2 × 10^9^ CFU/capsule) before meals for 3 weeks (*n* = 12) or placebo (*n* = 10). The study involved individuals with CD who were not adhering to a GFD. Individuals consuming at least 12 g of gluten per day were considered for the study. Inflammatory markers associated with CD were evaluated, and the baseline ratio for serum concentrations of IgA tTG had a significant reduction (*p *= 0.055) in individuals receiving *B. infantis* NLS super strain compared to those on placebo. A similar trend was observed for IgA DGP, although the reduction was not statistically significant (*p *= 0.181). As for the results of inflammatory mediators, the baseline serum cytokine profile with a Th1 bias shown in plasma did not change significantly in the analysis within the groups after both treatments.

Similarly, no significant changes were detected in the tested chemokine serum concentration. However, a significant increase in a chemokine agonist (MIP‐1β) was observed, which might be linked to the improvement of symptoms in individuals using the probiotic. It was also suggested that using a CCR5 agonist, a key receptor involved in controlling the migration of monocytes/macrophages and T lymphocytes (such as MIP‐1β), in conjunction with high‐dose oral antigen, might help establish a balance of GALT cytokines during ongoing autoimmune disease, promoting an anti‐inflammatory state and reducing autoreactivity [58]. However, further studies are needed to evaluate this issue.

Translating findings from preclinical studies into clinical outcomes present several challenges, particularly regarding the survival and functional activity of strains through the gastrointestinal tract, as well as by the physiological differences among individuals with CD. For example, gastric pH, transit time, and local inflammation may influence whether viable strains reach the small intestine in sufficient numbers to exert their effects [62]. Additionally, the probiotic delivery form (e.g., capsules, fermented foods, sachets) significantly affects bacterial viability and colonization success. The host's existing microbiota also plays a critical role in determining whether probiotic strains will persist and interact with the immune system effectively [58].

Although in vitro assays and animal models have provided important mechanistic insights, the translation of these findings to human clinical settings faces notable challenges. As highlighted by Laparra et al. [13] and Lindfors et al. [5], both animal models and epithelial cell lines, while informative, fail to fully replicate the multifactorial and heterogeneous nature of coeliac disease in humans. Differences in microbial colonization, immune responses, strain‐specific viability, and host variability, such as genetic background and dietary patterns, can influence the outcomes of probiotic interventions. Although these findings are promising, it is critical to underscore that probiotics should rather be viewed as complementary tools within a broader dietary and therapeutic strategy. Therefore, clinical validation through well‐controlled human trials remains essential to confirm the efficacy, safety, and practical application of these findings in CD management.

#### 4.2.4 Alteration in Intestinal Microbiome

Smecuol et al. [63] examined the impact of *B. infantis* on individuals with treated CD through a randomized, crossover, double‐blind, placebo‐controlled trial involving symptomatic adults who had been on a GFD for a minimum of 2 years. The study cohort comprised 12 adults (>18 years old) diagnosed with CD according to current criteria while adhering to a gluten‐containing diet. The participants were directed to consume two capsules of *B. infantis* NLS‐SS containing 2 × 10^9^ CFU/capsule three times a day, or placebo, for 3 weeks. The research results indicate no differences between placebo and *B. infantis* NLS‐SS treatments in the general population. Nevertheless, when analyzing patients with a higher clinical burden, it was observed that *B. infantis* significantly improved specific celiac symptom scores compared to those receiving a placebo (*p *< 0.03). These individuals considered themselves strictly compliant with the GFD.

To investigate potential mechanisms underlying the symptomatic improvement with *B. infantis*, fecal microbiota profiles were examined through 16S rRNA sequencing [63]. The administration of *B. infantis* induced alterations in the fecal microbiota profile, suggesting a modification in gut microbiota composition during short‐term *B. infantis* treatment. Moreover, the abundance of *B. infantis* increased during probiotic treatment, and higher levels of *B. longum* were observed in individuals with elevated symptom scores, supporting the idea that bifidobacteria can influence gut microbiota, potentially contributing to the observed beneficial response in this subgroup [63]. However, future studies should explore whether *B. infantis* affects the microbiota in the small intestine and how this correlates with sustained symptom improvement and intestinal morphology, especially over a more extended treatment period involving a larger participant pool.

Francavilla et al. [64] conducted a randomized, double‐blind, placebo‐controlled study involving adult volunteers (>18 years old) diagnosed with CD who had adhered to a strict GFD for at least 2 years. All participants underwent a comprehensive clinical evaluation, including assessments of gastrointestinal function, quality of life, CD serology, and standard laboratory parameters. Only individuals with negative celiac antibodies and strict adherence to the GFD were included. A total of 109 individuals were randomly assigned to either the probiotics group (*n* = 54) or the placebo group (*n* = 55). The probiotic formulation consisted of five strains of lactic acid bacteria: *L. casei* LMG 101/37 P‐17504 (5 × 10^9^ CFU/sachet), *L. plantarum* CECT 4528 (5 × 10^9^ CFU/sachet), *B. animalis* subsp. *lactis* Bi1 LMG P‐17502 (10 × 10^9^ CFU/sachet), *B. breve* Bbr8 LMG P‐17501 (10 × 10^9^ CFU/sachet), and *B. breve* Bl10 LMG P‐17500 (10 × 10^9^ CFU/sachet). The probiotics were administered daily in sachet form for 6 weeks. At the end of the intervention, significant improvements were observed in the probiotics group compared to the placebo group. Scores on the Irritable Bowel Syndrome Severity Scoring System (IBS‐SSS) and the Gastrointestinal Symptom Rating Scale (GSRS) significantly decreased. Additionally, treatment success was higher in the probiotics group than in the placebo group (15.3% vs. 3.8%, *p* < 0.04). Increases in presumed lactic acid bacteria, *Staphylococcus*, and *Bifidobacterium* were observed in participants receiving the probiotic treatment. No adverse events were reported. However, no clinically significant differences in stool appearance were noted between the groups. As a limitation, the study did not include intestinal biopsies, which could have provided further insights into the relationship between persistent symptoms and mucosal inflammation.

The study by Harnett et al. [62] enrolled individuals diagnosed with CD who continued to present symptoms despite reporting strict adherence to a GFD over the previous 12 months. Eligible participants were between 18 and 70 years old, had a confirmed CD diagnosis by duodenal biopsy for at least 1 year, and had followed a strict GFD for a minimum of 12 months. A total of 45 individuals were randomly assigned to receive either a probiotic formulation (*n* = 23) or a placebo (*n* = 22). During the intervention phase, three participants dropped out: two from the placebo group due to difficulties in adhering to the study protocol, and one from the probiotics group due to worsened constipation attributed to the study medication. Consequently, complete data were obtained from 42 participants.

This was the first human study reported to assess the microbiological effects of a gram‐positive multispecies probiotic in individuals with CD [62]. The primary outcome measured the efficacy of the probiotic formula in altering fecal microbiota counts between the study's initiation and week 12. However, the results did not reveal significant differences between the active and placebo groups regarding the primary outcome measure and fecal microbiota count. Alternative outcomes could be achieved by increasing the probiotic dose, extending the study duration, and preferably by analyzing results obtained in at least two different laboratories. Another hypothesis is that probiotic species may not have survived the physiological environment of the upper gastrointestinal tract. It is crucial for viable bacteria counts to reach the small and large intestines, where they can exert their beneficial effects [65]. The survival of probiotic microbes during gastrointestinal transit has been linked to the buffering capacity of foods, suggesting that non‐enteric coated bacterial probiotic products should be consumed with or immediately before a meal, ideally one containing some fats [66]. Harnett et al. [62] also did not control for fat content in this study, a factor that should be considered in future research.

## Concluding Remarks

*Bifidobacterium* species present promising characteristics as a potential adjunctive strategy in CD management. However, the effects seem strain‐specific and impacted by various factors, such as delivery form, host conditions, and use in combination with other strains. According to the analysis of the articles included in this review, Bifidobacterium spp. can modulate mechanisms related to gluten‐induced cytotoxicity and inflammation, which led us to identify six potential mechanisms of action: (1) induction of COX‐1 expression in Caco‐2 cells and reduction of COX‐2 expression; (2) decreased zonulin activation; (3) hydrolysis of gliadin‐derived peptides; (4) inhibition of CXCR3 mRNA expression; (5) reduction of TNF‐α, NF‐kB, and IL‐1β production; and (6) modification of the gut microbiota. Few of these studies were performed or translated to in vivo, but to date, it seems unclear if *Bifidobacterium* spp. can help to prevent the onset of CD and manage CD or if GFD can be disregarded. Future research should focus on clarifying these gaps.

To advance the clinical application of *Bifidobacterium*‐based interventions for CD, the following future research priorities are recommended, such as: Conduct long‐term RCTs including histological and symptom‐based endpoints in individuals with CD; standardize probiotic formulations, investigate synergistic effects of multi‐strain combinations, especially those involving both *Bifidobacterium* and other beneficial genera and explore host–microbe interactions through advanced in vivo and ex vivo CD models to clarify mechanisms and patient‐specific responses.

## Author Contribution

T.C.S.—Investigation, data interpretation, analysis and draft writing; I.M. —interpretation of data and review of the manuscript; N.R.C.F.—interpretation of data and review of the manuscript; T.C.P.—interpretation of data and review of the manuscript; M.M.—interpretation of data and review of the manuscript; G.S.H.—interpretation of data and review of the manuscript; A.B.N.—interpretation of data and review of the manuscript; S.V.—project administration, resources, conceptualization, supervision, data interpretation, editing and revision.

## Conflicts of Interest

The authors reported no potential conflict of interest.

## Data Availability

The data that support the findings of this study are available from the corresponding author, Silvani Verruck, upon request.

## References

[1] M. K. Kowalski , D. Domżał‐Magrowska , and E. Małecka‐Wojciesko , “Celiac Disease—Narrative Review on Progress in Celiac Disease,” Foods 14 (2025): 959. 40231983 10.3390/foods14060959 PMC11941517

[2] G. Caio , U. Volta , A. Sapone , et al., “Celiac Disease: A Comprehensive Current Review,” BMC Medicine 17 (2019): 142. 31331324 10.1186/s12916-019-1380-z PMC6647104

[3] E. Ferrari , R. Monzani , V. Saverio , et al., “Probiotics Supplements Reduce ER Stress and Gut Inflammation Associated With Gliadin Intake in a Mouse Model of Gluten Sensitivity,” Nutrients 13 (2021): 1221. 33917155 10.3390/nu13041221 PMC8067866

[4] E. Khalkhal , M. Rezaei‐Tavirani , N. Asri , et al., “Introducing New Potential Biomarkers for Celiac Disease Among the Genes Extracted From General Databases,” Middle East Journal of Digestive Disease 14 (2022): 192–199. 10.34172/mejdd.2022.272 PMC9489306 36619141

[5] K. Lindfors , T. Blomqvist , K. Juuti‐Uusitalo , et al., “Live Probiotic *Bifidobacterium lactis* Bacteria Inhibit the Toxic Effects Induced by Wheat Gliadin in Epithelial Cell Culture,” Clinical and Experimental Immunology 152 (2008): 552–558. 18422736 10.1111/j.1365-2249.2008.03635.x PMC2453197

[6] A. Taetzsch , S. K. Das , C. Brown , A. Krauss , R. E. Silver , and S. B. Roberts , “Are Gluten‐Free Diets More Nutritious? An Evaluation of Self‐Selected and Recommended Gluten‐Free and Gluten‐Containing Dietary Patterns,” Nutrients 10 (2018): 1881. 30513876 10.3390/nu10121881 PMC6317051

[7] V. Segura , Á. Ruiz‐Carnicer , C. Sousa , and M. L. de Moreno , “New Insights Into Non‐Dietary Treatment in Celiac Disease: Emerging Therapeutic Options,” Nutrients 13 (2021): 2146. 34201435 10.3390/nu13072146 PMC8308370

[8] S. Norouzbeigi , L. Vahid‐Dastjerdi , R. Yekta , S. Sohrabvandi , F. Zendeboodi , and A. M. Mortazavian , “Celiac Therapy by Administration of Probiotics in Food Products: A Review,” Current Opinion in Food Science 32 (2020): 58–66.

[9] R. Chibbar and L. A. Dieleman , “The Gut Microbiota in Celiac Disease and Probiotics,” Nutrients 11 (2019): 2375. 31590358 10.3390/nu11102375 PMC6836185

[10] A. Orlando , M. Linsalata , G. Bianco , et al., “ *Lactobacillus rhamnosus* GG Protects the Epithelial Barrier of Wistar Rats From the Pepsin‐Trypsin‐Digested Gliadin (PTG)‐Induced Enteropathy,” Nutrients 10 (2018): 1698. 30405050 10.3390/nu10111698 PMC6265991

[11] M. Olivares , M. Laparra , and Y. Sanz , “Oral Administration of *Bifidobacterium longum* CECT 7347 Modulates Jejunal Proteome in an In Vivo Gliadin‐Induced Enteropathy Animal Model,” Proteomics 77 (2012): 310–320. 23023000 10.1016/j.jprot.2012.09.005

[12] R. D'Arienzo , R. Stefanile , F. Maurano , et al., “Immunomodulatory Effects of *Lactobacillus casei* Administration in a Mouse Model of Gliadin‐Sensitive Enteropathy,” Scandinavian Journal of Immunology 74 (2011): 335–341. 21615450 10.1111/j.1365-3083.2011.02582.x

[13] J. M. Laparra , M. Olivares , O. Gallina , and Y. Sanz , “ *Bifidobacterium longum* CECT 7347 Modulates Immune Responses in a Gliadin‐Induced Enteropathy Animal Model,” PLOS ONE 7 (2012): 30744. 10.1371/journal.pone.0030744 PMC3277586 22348021

[14] M. Beikmohammadi , S. Halimi , N. A. Fard , and W. Wen , “Therapeutic Potential of Probiotics: A Review of Their Role in Modulating Inflammation,” Probiotics and Antimicrobial Proteins (2025), 10.1007/s12602-025-10609-z. 40465090

[15] M. Medina , G. De Palma , C. Ribes‐Koninckx , M. Calabuig , and Y. Sanz , “ *Bifidobacterium* Strains Suppress In Vitro the Pro‐Inflammatory Milieu Triggered by the Large Intestinal Microbiota of Coeliac Patients,” Journal of Inflammation 5 (2008): 19. 18980693 10.1186/1476-9255-5-19 PMC2640389

[16] A. Ramesh , D. Srinivasan , R. Subbarayan , et al., “Enhancing Colorectal Cancer Treatment: The Role of *Bifidobacterium* in Modulating Gut Immunity and Mitigating Capecitabine‐Induced Toxicity,” Molecular Nutrition & Food Research 69 (2025): 70023, 10.1002/mnfr.70023. 40109200

[17] F. A. C. Martinez , E. M. Balciunas , A. Converti , P. D. Cotter , and R. P. de Souza Oliveira , “Bacteriocin Production by *Bifidobacterium* spp. A Review,” Biotechnology Advances 31 (2013): 482–488. 23384878 10.1016/j.biotechadv.2013.01.010

[18] M.‐E. Boyte , N. Akhtar , B. Koshy , and A. L. Roe , “A Review of Probiotic Ingredient Safety Supporting Monograph Development Conducted by the United States Pharmacopeia (USP),” Journal of Dietary Supplements 22 (2025): 123–161. 38356247 10.1080/19390211.2024.2314488

[19] S. P. Borriello , W. P. Hammes , W. Holzapfel , et al., “Safety of Probiotics That Contain Lactobacilli or Bifidobacteria,” Clinical Infectious Diseases 36 (2003): 775–780. 12627362 10.1086/368080

[20] J.‐J. Dugoua , M. Machado , X. Zhu , X. Chen , G. Koren , and T. R. Einarson , “Probiotic Safety in Pregnancy: A Systematic Review and Meta‐Analysis of Randomized Controlled Trials of Lactobacillus, Bifidobacterium, and Saccharomyces spp,” Journal of Obstetrics and Gynaecology Canada 31 (2009): 542–552. 19646321 10.1016/S1701-2163(16)34218-9

[21] S.‐P. Shen , H.‐C. Lin , J.‐F. Chen , et al., “Assessment of the Safety and Gut Microbiota Modulation Ability of an Infant Formula Containing Bifidobacterium Animalis ssp. Lactis CP‐9 or Lactobacillus Salivarius AP‐32 and the Effects of the Formula on Infant Growth Outcomes: Insights From a Four‐Month Clinical Study in Infants Under Two Months Old,” BMC Pediatrics 24 (2024): 840. 39731060 10.1186/s12887-024-05289-7 PMC11674581

[22] H. Tsai , Y. Wang , C. Liao , et al., “Safety and the Probiotic Potential of Bifidobacterium Animalis CP‐9,” Journal of Food Science 87 (2022): 2211–2228. 35347713 10.1111/1750-3841.16129

[23] S. J. Allen , S. Jordan , M. Storey , et al., “Dietary Supplementation with Lactobacilli and Bifidobacteria Is Well Tolerated and Not Associated With Adverse Events During Late Pregnancy and Early Infancy,” Journal of Nutrition 140 (2010): 483–488. 20089774 10.3945/jn.109.117093

[24] M. Kujawska , K. Neuhaus , C. Huptas , et al., “Exploring the Potential Probiotic Properties of *Bifidobacterium breve* DSM 32583—A Novel Strain Isolated From Human Milk,” Probiotics and Antimicrobial Proteins (2024), 10.1007/s12602-024-10346-9. PMC12634702 39287748

[25] C. B. Wong , T. Odamaki , and J. Xiao , “Beneficial Effects of *Bifidobacterium longum* subsp. *Longum* BB536 on Human Health: Modulation of Gut Microbiome as the Principal Action,” Journal of Functional Foods 54 (2019): 506–519.

[26] M. J. Kim , S. Ku , S. Y. Kim , et al., “Safety Evaluations of *Bifidobacterium bifidum* BGN4 and *Bifidobacterium longum* BORI,” International Journal of Molecular Sciences 19 (2018): 1422. 29747442 10.3390/ijms19051422 PMC5983828

[27] D. A. Leffler , M. Dennis , B. Hyett , E. Kelly , D. Schuppan , and C. P. Kelly , “Etiologies and Predictors of Diagnosis in Nonresponsive Celiac Disease,” Clinical Gastroenterology and Hepatology 5 (2007): 445–450. 17382600 10.1016/j.cgh.2006.12.006

[28] G. De Palma , J. Cinova , R. Stepankova , L. Tuckova , and Y. Sanz , “Pivotal Advance: Bifidobacteria and Gram‐negative Bacteria Differentially Influence Immune Responses in the Proinflammatory Milieu of Celiac Disease,” Journal of Leukocyte Biology 87 (2009): 765–778. 20007908 10.1189/jlb.0709471

[29] G. Barbara , M. R. Barbaro , D. Fuschi , et al., “Inflammatory and Microbiota‐Related Regulation of the Intestinal Epithelial Barrier,” Frontiers in Nutrition 8 (2021): 718356. 34589512 10.3389/fnut.2021.718356 PMC8475765

[30] T. Otani and M. Furuse , “Tight Junction Structure and Function Revisited,” Trends in Cell Biology 30 (2020): 805–817. 33097373 10.1016/j.tcb.2020.10.001

[31] C. Zihni , C. Mills , K. Matter , and M. S. Balda , “Tight Junctions: From Simple Barriers to Multifunctional Molecular Gates,” Nature Reviews Molecular Cell Biology 17 (2016): 564–580. 27353478 10.1038/nrm.2016.80

[32] M. A. Garcia , W. J. Nelson , and N. Chavez , “Cell–Cell Junctions Organize Structural and Signaling Networks,” Cold Spring Harbor Perspectives in Biology 10 (2018): a029181. 28600395 10.1101/cshperspect.a029181 PMC5773398

[33] N. Wibbe and K. Ebnet , “Cell Adhesion at the Tight Junctions: New Aspects and New Functions,” Cells 12 (2023): 2701. 38067129 10.3390/cells12232701 PMC10706136

[34] A. Fasano , “Intestinal Permeability and Its Regulation by Zonulin: Diagnostic and Therapeutic Implications,” Clinical Gastroenterology and Hepatology 10 (2012): 1096–1100. 22902773 10.1016/j.cgh.2012.08.012 PMC3458511

[35] C. Sturgeon and A. Fasano , “Zonulin, A Regulator of Epithelial and Endothelial Barrier Functions, and Its Involvement in Chronic Inflammatory Diseases,” Tissue Barriers 4 (2016): 1251384. 10.1080/21688370.2016.1251384 PMC5214347 28123927

[36] A. Balakireva and A. Zamyatnin , “Properties of Gluten Intolerance: Gluten Structure, Evolution, Pathogenicity and Detoxification Capabilities,” Nutrients 8 (2016): 644. 27763541 10.3390/nu8100644 PMC5084031

[37] I. Caputo , A. Secondo , M. Lepretti , et al., “Gliadin Peptides Induce Tissue Transglutaminase Activation and ER‐stress through Ca2+ Mobilization in Caco‐2 Cells,” PLOS ONE 7 (2012): 45209. 10.1371/journal.pone.0045209 PMC3458012 23049776

[38] D. Dewar , S. P. Pereira , and P. J. Ciclitira , “The Pathogenesis of Coeliac Disease,” International Journal of Biochemistry & Cell Biology 36 (2004): 17–24. 14592529 10.1016/s1357-2725(03)00239-5

[39] M. Primec , M. Klemenak , D. Di Gioia , et al., “Clinical Intervention Using *Bifidobacterium* Strains in Celiac Disease Children Reveals Novel Microbial Modulators of TNF‐α and Short‐Chain Fatty Acids,” Clinical Nutrition 38 (2019): 1373–1381. 29960810 10.1016/j.clnu.2018.06.931

[40] L. Jelínková , L. Tučková , J. Cinová , Z. Flegelová , and H. Tlaskalová‐Hogenová , “Gliadin Stimulates Human Monocytes to Production of IL‐8 and TNF‐α Through a Mechanism Involving NF‐κB,” FEBS Letters 571 (2004): 81–85. 15280021 10.1016/j.febslet.2004.06.057

[41] J. M. Laparra and Y. Sanz , “Bifidobacteria Inhibit the Inflammatory Response Induced by Gliadins in Intestinal Epithelial Cells via Modifications of Toxic Peptide Generation During Digestion,” Journal of Cellular Biochemistry 109 (2010): 801–807. 20052669 10.1002/jcb.22459

[42] A. A. Hajeyah , W. J. Griffiths , Y. Wang , A. J. Finch , and V. B. O'Donnell , “The Biosynthesis of Enzymatically Oxidized Lipids,” Frontiers in Endocrinology (Lausanne) 11 (2020): 591819, 10.3389/fendo.2020.591819. PMC7711093 33329396

[43] M. Kawamura , H. Inaoka , S. Obata , and Y. Harada , “Why Do a Wide Variety of Animals Retain Multiple Isoforms of Cyclooxygenase?,” Prostaglandins & Other Lipid Mediators 109‐111 (2014): 14–22. 10.1016/j.prostaglandins.2014.03.002 24721150

[44] M. De Angelis , A. Cassone , C. G. Rizzello , et al., “Mechanism of Degradation of Immunogenic Gluten Epitopes From *Triticum Turgidum* L. var. *Durum* by Sourdough Lactobacilli and Fungal Proteases,” Applied and Environmental Microbiology 76 (2010): 508–518. 19948868 10.1128/AEM.01630-09 PMC2805216

[45] Y. E. Dunaevsky , V. F. Tereshchenkova , M. A. Belozersky , I. Y. Filippova , B. Oppert , and E. N. Elpidina , “Effective Degradation of Gluten and Its Fragments by Gluten‐Specific Peptidases: A Review on Application for the Treatment of Patients With Gluten Sensitivity,” Pharmaceutics 13 (2021): 1603. 34683896 10.3390/pharmaceutics13101603 PMC8541236

[46] F. Cristofori , V. N. Dargenio , C. Dargenio , V. L. Miniello , M. Barone , and R. Francavilla , “Anti‐Inflammatory and Immunomodulatory Effects of Probiotics in Gut Inflammation: A Door to the Body,” Frontiers in Immunology 12 (2021): 578386, 10.3389/fimmu.2021.578386. 33717063 PMC7953067

[47] A. Giorgi , R. Cerrone , D. Capobianco , et al., “A Probiotic Preparation Hydrolyzes Gliadin and Protects Intestinal Cells from the Toxicity of Pro‐Inflammatory Peptides,” Nutrients 12 (2020): 495. 32075195 10.3390/nu12020495 PMC7071319

[48] K. M. Lammers , R. Lu , J. Brownley , et al., “Gliadin Induces an Increase in Intestinal Permeability and Zonulin Release by Binding to the Chemokine Receptor CXCR3,” Gastroenterology 135 (2008): 3. 10.1053/j.gastro.2008.03.023 PMC2653457 18485912

[49] D. Talipova , A. Smagulova , and D. Poddighe , “Toll‐Like Receptors and Celiac Disease,” International Journal of Molecular Sciences 24 (2022): 265. 36613709 10.3390/ijms24010265 PMC9820541

[50] C. G. Rizzello , M. De Angelis , R. Di Cagno , et al., “Highly Efficient Gluten Degradation by Lactobacilli and Fungal Proteases During Food Processing: New Perspectives for Celiac Disease,” Applied and Environmental Microbiology 73 (2007): 4499–4507. 17513580 10.1128/AEM.00260-07 PMC1932817

[51] M. De Angelis , L. Vannini , R. Di Cagno , et al., “Salivary and Fecal Microbiota and Metabolome of Celiac Children Under Gluten‐Free Diet,” International Journal of Food Microbiology 239 (2016): 125–132. 27452636 10.1016/j.ijfoodmicro.2016.07.025

[52] A. Veres‐Székely , C. Szász , D. Pap , B. Szebeni , P. Bokrossy , and Á. Vannay , “Zonulin as a Potential Therapeutic Target in Microbiota‐Gut‐brain Axis Disorders: Encouraging Results and Emerging Questions,” International Journal of Molecular Sciences 24 (2023): 7548. 37108711 10.3390/ijms24087548 PMC10139156

[53] H. Zhao , L. Wu , G. Yan , et al., “Inflammation and Tumor Progression: Signaling Pathways and Targeted Intervention,” Signal Transduction and Targeted Therapy 6 (2021): 263. 34248142 10.1038/s41392-021-00658-5 PMC8273155

[54] A. Fasano and T. Shea‐Donohue , “Mechanisms of Disease: The Role of Intestinal Barrier Function in the Pathogenesis of Gastrointestinal Autoimmune Diseases,” Nature Clinical Practice Gastroenterology and Hepatology 2 (2005): 416–422. 10.1038/ncpgasthep0259 16265432

[55] A. L. Hart , K. Lammers , P. Brigidi , et al., “Modulation of Human Dendritic Cell Phenotype and Function by Probiotic Bacteria,” Gut 53 (2004): 1602–1609. 15479680 10.1136/gut.2003.037325 PMC1774301

[56] B. Rowshanravan , N. Halliday , and D. M. Sansom , “CTLA‐4: A Moving Target in Immunotherapy,” Blood 131 (2018): 58–67. 29118008 10.1182/blood-2017-06-741033 PMC6317697

[57] R. Štěpánková , O. Kofroňová , L. Tučková , H. Kozáková , J. J. Cebra , and H. Tlaskalová‐ Hogenová , “Experimentally Induced Gluten Enteropathy and Protective Effect of Epidermal Growth Factor in Artificially Fed Neonatal Rats,” Journal of Pediatric Gastroenterology and Nutrition 36 (2003): 96–104. 12500003 10.1097/00005176-200301000-00018

[58] M. C. Maiuri , D. De Stefano , G. Mele , et al., “Nuclear Factor κB Is Activated in Small Intestinal Mucosa of Celiac Patients,” Journal of Molecular Medicine 81 (2003): 373–379. 12743709 10.1007/s00109-003-0440-0

[59] A. Izcue , J. L. Coombes , and F. Powrie , “Regulatory Lymphocytes and Intestinal Inflammation,” Annual Review of Immunology 27 (2009): 313–338. 10.1146/annurev.immunol.021908.132657 19302043

[60] N. E. C. de Almeida , F. G. Esteves , J. R. A. dos Santos‐Pinto , et al., “Digestion of Intact Gluten Proteins by *Bifidobacterium* Species: Reduction of Cytotoxicity and Proinflammatory Responses,” Journal of Agricultural and Food Chemistry 68 (2020): 4485–4492. 32195585 10.1021/acs.jafc.0c01421

[61] E. Smecuol , H. J. Hwang , E. Sugai , et al., “Exploratory, Randomized, Double‐Blind, Placebo‐Controlled Study on the Effects of *Bifidobacterium infantis* Natren Life Start Strain Super Strain in Active Celiac Disease,” Journal of Clinical Gastroenterology 47 (2013): 139–147. 23314670 10.1097/MCG.0b013e31827759ac

[62] J. Harnett , S. P. Myers , and M. Rolfe , “Probiotics and the Microbiome in Celiac Disease: A Randomised Controlled Trial,” Evidence‐Based Complement Alternative Medicine 2016 (2016): 9048574, 10.1155/2016/9048574. PMC4972910 27525027

[63] E. Smecuol , M. Constante , M. P. Temprano , et al., “Effect of *Bifidobacterium infantis* NLS Super Strain in Symptomatic Coeliac Disease Patients on Long‐Term Gluten‐Free Diet—An Exploratory Study,” Beneficial Microbes 11 (2020): 527–534. 33032471 10.3920/BM2020.0016

[64] R. Francavilla , M. Piccolo , A. Francavilla , et al., “Clinical and Microbiological Effect of a Multispecies Probiotic Supplementation in Celiac Patients With Persistent IBS‐type Symptoms,” Journal of Clinical Gastroenterology 53 (2019): e117–e125. 29688915 10.1097/MCG.0000000000001023 PMC6382041

[65] S. Stummer , S. Salar‐Behzadi , F. M. Unger , S. Oelzant , M. Penning , and H. Viernstein , “Application of Shellac for the Development of Probiotic Formulations,” Food Research International 43 (2010): 1312–1320.

[66] T. Tompkins , I. Mainville , and Y. Arcand , “The Impact of Meals on a Probiotic During Transit through a Model of the Human Upper Gastrointestinal Tract,” Beneficial Microbes 2 (2011): 295–303. 22146689 10.3920/BM2011.0022

[67] N. Ramedani , A. Seidita , N. Asri , et al., “The Gliadin Hydrolysis Capacity of *B. longum*, *L. acidophilus*, and *L. plantarum* and Their Protective Effects on Caco‐2 Cells Against Gliadin‐Induced Inflammatory Responses,” Nutrients 15 (2023): 2769. 37375673 10.3390/nu15122769 PMC10301210

[68] N. Wang , Z. Pei , H. Wang , J. Zhao , and W. Lu , “ *Bifidobacterium longum* Ameliorates Intestinal Inflammation and Metabolic Biomarkers in Mice Fed a High‐Fat Diet with Gliadin by Indoleacrylic Acid,” Probiotics and Antimicrobial Proteins (2025), 10.1007/s12602-025-10486-6. 39982644

